# Noradrenergic Locus Coeruleus Hyperactivity Alters Attention in Cognitively Healthy Older Adults

**DOI:** 10.1093/geroni/igaf122.3817

**Published:** 2025-12-31

**Authors:** Joshua Senior, Andy Kim, Santiago Morales, Mara Mather

**Affiliations:** Massachusetts General Hospital, Harvard Medical School, Boston, Massachusetts, United States; Leonard Davis School of Gerontology, University of Southern California, Los Angeles, California, United States; University of Southern California, Los Angeles, California, United States; Leonard Davis School of Gerontology, University of Southern California, Los Angeles, California, United States

## Abstract

Animal models of Alzheimer’s disease have shown that neuropathology in the locus coeruleus (LC) leads to increased neuronal firing, while human studies demonstrate that age-related dysregulation of the LC-noradrenaline (LC-NA) system is associated with cognitive decline. However, due to limitations in directly measuring LC function in vivo, it remains unclear whether age-related alterations in humans reflect tonic LC-NA system hyperactivity. In this study, we tested the hypothesis that cognitively healthy older adults sustain tonic LC hyperactivity, by acquiring electrophysiological, pupillometric, and behavioral measures during a passive and active auditory oddball paradigm. We capitalized on the LC-NA system’s role in arousal regulation and manipulated state arousal using the threat of electric shock. We hypothesized that if older adults maintain elevated LC activity compared with young adults, task-evoked noradrenergic responses would be less responsive to arousal in older adults. Consistent with this hypothesis, arousal elicited weaker behavioral responses, pupil dilation responses, and P300 event-related potentials in older adults compared with young adults. Linear mixed models revealed an arousal by modality interaction and revealed that arousal differentially modulated attentional control to salient but task-irrelevant distractors between both age groups. Collectively, these findings support the hypothesis that aging is associated with tonic LC-NA system hyperactivity in humans, with downstream consequences for mechanisms of attentional control. These multimodal findings underscore the potential of non-invasive physiological markers to assess LC-NA system function throughout aging and identify individuals at elevated risk for neurodegenerative progression prior to the emergence of clinical biomarkers.

